# Impact of traumatic dental injuries on oral health-related quality of life of preschool children: A systematic review and meta-analysis

**DOI:** 10.1371/journal.pone.0172235

**Published:** 2017-02-28

**Authors:** Tássia Silvana Borges, Fabiana Vargas-Ferreira, Paulo Floriani Kramer, Carlos Alberto Feldens

**Affiliations:** Department of Pediatric Dentistry, Universidade Luterana do Brasil, Canoas, Brazil; University of Washington, UNITED STATES

## Abstract

**Background:**

Observational studies have suggested that traumatic dental injuries (TDI) can lead to pain, loss of function and esthetic problems, with physical, emotional and social consequences for children and their families. However, population-based studies that investigate the impact of TDI on oral health-related quality of life (OHRQoL) among preschool children are scarce and offer conflicting results. The aim of the systematic review and meta-analysis was to evaluate the impact of TDI on OHRQoL among preschool children (PROSPERO-CRD42015032513).

**Methods:**

An electronic search of six databases was performed in *PubMed (MEDLINE)*, *ISI Web of Science*, *Scopus*, *Science Direct*, *EMBASE* and *Google Scholar*, with no language or publication date restrictions. The eligibility criteria were TDI as the exposure variable, OHRQoL as the outcome and a population of children up to six years of age. RevMan software was used for data analysis. Results are expressed as odds ratios with 95% confidence intervals for the total score of the Early Childhood Oral Health Impact Scale (ECOHIS) as well as the scores of the Child Impact Section (CIS) and Family Impact Section (FIS). The random effect model was chosen and heterogeneity was evaluated using the I^2^ test.

**Results:**

2,013 articles were initially retrieved; 1,993 articles were excluded based on title and abstracts; 10 articles excluded after full-text analysis. Ten studies comprising a population of 7,461 preschool children were included in the systematic review and nine studies were included in the meta-analysis. TDI caused a negative impact on OHRQoL based on the overall ECOHIS (OR: 1.24; 95% CI: 1.08–1.43) and CIS (OR: 1.23; 95% CI: 1.07–1.41), but not the FIS (OR: 1.09; 95% CI: 0.90–1.32).

**Conclusions:**

TDI negatively impacted on OHRQoL of preschool children. The present findings indicate the need for TDI prevention and treatment programs in early childhood.

## Introduction

Traumatic dental injuries (TDI) in the primary dentition affects approximately one third of preschool children in different countries throughout the world and therefore represents one of the most prevalent outcomes in early childhood [1]. Observational studies have suggested that TDI, especially complicated crown fractures, luxations and avulsion, can lead to pain, loss of function and esthetic problems, with physical, emotional and social consequences for children and their families [2–5]. However, population-based studies that investigate the impact of TDI on oral health-related quality of life (OHRQoL) among preschool children are scarce and offer conflicting results [6–10].

Systematic reviews and meta-analyses are sources of information that summarize and organize data in the best possible form to allow the inference of results in the clearer and more decisive manner. In the field of oral health, such studies are developed to clarify risk factors for numerous outcomes, such as dental caries, erosion, periodontal disease, enamel defects and TDI [11–15]. However, no previous systematic review has investigated the impact of TDI on the OHRQoL of preschool children and their families. The clarification of this issue could contribute to the identification of oral problems that should be prioritized in the planning and definition of cost-effective prevention and treatment strategies on both the individual and collective levels [9,16]. Moreover, such information should be considered in the education and training of healthcare professionals, who should focus primarily on outcomes identified as clinically relevant.

Thus, the aim of the present study was to perform a systematic review and meta-analysis to investigate the impact of TDI in the primary dentition on the OHRQoL of preschool children and their families.

## Materials and methods

### Protocol and registry

The present systematic review was undertaken in accordance with the guidelines of the Preferred Reporting Items for Systematic Reviews and Meta-Analyses (PRISMA) [17] and the protocol was based on the PROSPERO registry (CRD 42015032513).

### Eligibility criteria

Epidemiological design (case-control, cross-sectional, cohort, randomized clinical trial) and systematic reviews with TDI as the exposure variable, OHRQoL as the outcome (determined using a validated questionnaire) and population of children up to six years of age were considered eligible. Articles with review, case reports, studies involving individuals aged older than six years and articles without the predefined outcome and exposure variables were excluded. No restrictions were imposed regarding language or year of publication.

### Search strategy and bibliographic sources

Two independent reviewers (TSB and FVF) searched the *PubMed (MEDLINE)*, *ISI Web of Science*, *Scopus*, *Science Direct*, *EMBASE* and *Google Scholar* (grey literature) databases from July to August 2015. Further searches were subsequently performed until June 2016. The following search strategy was used: [dental injuries (text words) OR dental trauma (text words) OR traumatic dental injuries (text words) OR traumatic dental (text words) OR tooth injuries (MeSH) OR tooth fractures (MeSH)] AND [quality of life (MeSH)] AND [preschool (MeSH) OR child (MeSH) OR children (text words)].

Manual searches were performed of the references lists of all articles retrieved for the identification of other relevant articles. All abstracts and titles were saved in a numbered, ordered fashion. The titles and abstracts were analyzed independently by each reviewer for the pre-selection of potentially eligible articles for systematic review and meta-analysis. The two reviewers then discussed the preselected articles and came to a consensus regarding which articles should be submitted to full-text analysis. For the determination of inter-examiner agreement, five potentially eligible articles were evaluated independently using the data extraction chart. The Kappa statistic was employed and demonstrated high inter-examiner agreement (K = 0.95).

### Data extraction

A data extraction spreadsheet was developed and the two researchers (TSB and FVF) collected the information independently. The information collected were year of publication, title of article, author’s name, study design (cross-sectional, cohort, case-control, randomized clinical trial or systematic review), location of study, language in which study was published, characteristics of the participants (sample size, age and sex), predictors evaluated, outcome and data analysis (tests employed, effect measures, confidence intervals and p-values).

### Quality of studies

The quality of the studies was evaluated by the same two independent reviewers using the Newcastle-Ottawa Scale for case-control and cohort studies [18]. Cross-sectional studies were evaluated using the modified Newcastle-Ottawa Scale [18], since there are no standardized, universally acceptable scales for this type of study design.

### Statistical methods and data synthesis

To estimate the impact of TDI on OHRQoL, the exposure variable was dichotomized (absence/presence of TDI). The Early Childhood Oral Health Impact Scale (ECOHIS) was used for the evaluation of the outcome, since it is the only validated instrument that assesses OHRQoL in preschool children. Among the 13 items on this scale, nine measure the impact of oral problems on the child [Child Impact Section (CIS)] and four measure the impact of the child’s oral problems on the family [Family Impact Section (FIS)]. The CIS has four domains (symptoms, function, psychological aspects and self-image/social interactions). The FIS has two domains (parental distress and family function). The score of the CIS ranges from 0 to 36 points and the score on the FIS ranges from 0 to 16 points. The response options are 1) never, 2) hardly ever, 3) occasionally, 4) often, 5) very often and 6) “Don’t know”. The cutoff point was established as never/rarely (absence of impact) and at least one response of sometimes, often or every day/almost every day (presence of impact). This cutoff point is commonly employed in studies and was also established by the authors of the ECOHIS [19]. The total and subscale (CIS and FIS) scores were considered. All classifications were predefined for standardization and subsequent meta-analysis. The authors of articles included in the systematic review were contacted to provide further information based on these definitions when needed.

RevMan software (V5.2) was used to analyze the data for heterogeneity and produce a graphical display of results. The number of events (impact on OHRQoL) and number of individuals with and without TDI were selected. Effect measures [odds ratio (OR)] and 95% confidence intervals (CIs) were estimated for the total ECOHIS score as well as the scores of the CIS and FIS. A subgroup meta-analysis was conducted taking into account the two designs (case-control and cross-sectional). For both forest plots, 95% CIs and p-values were calculated. Heterogeneity among the results of studies and the quantification of inconsistency were evaluated using the I^2^ test [20]. The random effect model was used for meta-analysis in all cases due to the fact that, when studies are gathered from published literature, the random effects model is generally a more plausible match [21].

## Results

**Fig 1:**
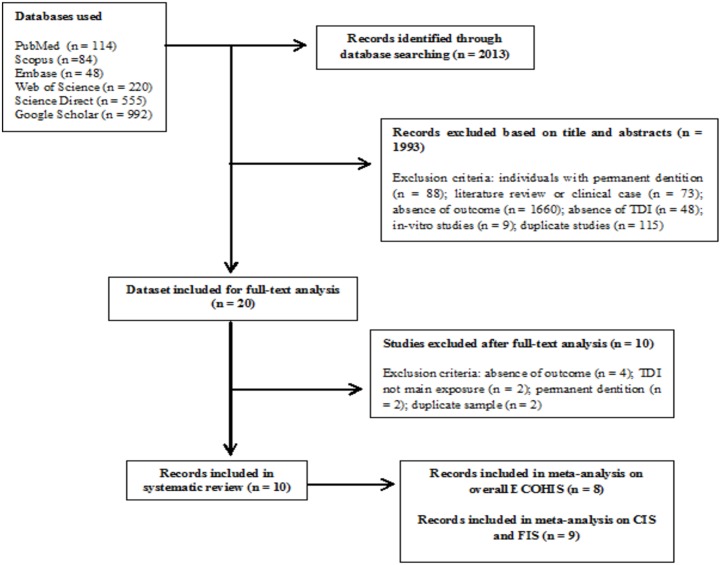
Flowchart for study collection showing number of studies identified, screened, eligible and included in systematic review and meta-analysis.

Fig 1 displays the flowchart describing the number of articles identified in each step of the study. The search strategy led to the initial retrieval of 2013 articles, 1993 of which were excluded during the screening of the titles and abstracts. Twenty articles were submitted to full-text analysis, ten of which were excluded for the following reasons: the outcome was not OHRQoL or the subscales (n = 4); TDI was not the main exposure (n = 2); the age range was inappropriate (n = 2); and duplicate samples were used (n = 2). Thus, the final sample of the present systematic review included ten articles comprising 7,461 preschool children. Authors were contacted to provide missing information that was important to the purposes of the present investigation. For meta-analysis, two articles were excluded from the analysis of the effect on overall OHRQoL and one was excluded for the analysis of the impact on the FIS and CIS, as incomplete data were acquired even after various contacts with the authors. Thus, meta-analysis was performed with eight articles for the overall ECOHIS and nine articles for the CIS and FIS.

Table 1 summarizes the main characteristics of each study analyzed in the present systematic review. All studies were conducted in Brazil and all measured the outcome using the ECOHIS questionnaire. Age of the preschool children ranged from one to six years. The criteria used for the diagnosis of TDI were those proposed by Andreasen et al. (2007) [22] [9, 23–28] (n = 7), Glendor et al. (1996) [29] [3,4] (n = 2) and O’Brien (1994) [30] [31] (n = 1). Most studies employed a cross-sectional design [3,4,9,23–26,31] (n = 8) and the remaining two were case-control studies [27,28].

**Table 1:** Characteristics of studies included in systematic review.

Author(s)and language	Year	Country	Sample; age (years)	Study design	Main exposure definition	Outcome definition	Other measures	Effect size and crude or adjusted association results with 95% CI
Aldrigui et al.[3] English	2011	Brazil	260; 2–5	Cross-sectional	TDI (Glendor)	Scores of OHRQoL (ECOHIS)	Age, Gender, Dental caries, Anterior Open Bite, Types of TDI	**Impact on overall ECOHIS** Uncomplicated TDI- RR: 0.89 (0.66–1.20); Complicated TDI- RR: 1.90 (1.38–2.62)
Viegas et al.[23] English	2012	Brazil	388; 5–6	Cross-sectional	TDI (Andreasen)	Scores of OHRQoL (ECOHIS)	Gender, No. of people in household, Household income, Social Vulnerability Index, Parents’/caregivers’ schooling, Lip competence, Overjet, Anterior crossbite and open bite, Overbite, Dental caries; Enamel defects	**Impact on CIS** Presence of TDI-PR: 1.15 (0.92–1.42); **Impact on FIS** Presence TDI -PR: 1.28 (0.95–1.71)
Siqueira et al.[24] English	2013	Brazil	814; 3–5	Cross-sectional	TDI (Andreasen)	OHRQoL as a dichotomous variable (ECOHIS)	Gender, Number of residents in home, Household income, Parent/caregiver’s schooling, Parent/caregiver’s assessment of child’s oral health, Parent/caregiver’s assessment of child’s general health, TDI and types, Number of teeth affected by TDI Toothache, Visits to dentist, Type of preschool	**Impact on CIS** Presence TDI -PR: 1.10 (0.89–1.36); **Impact on FIS** Presence TDI—PR: 1.12 (0.79–1.31)
Kramer et al.[9]English	2013	Brazil	1036; 2–5	Cross-sectional	TDI (Andreasen)	OHRQoL as a dichotomous variable (ECOHIS)	Gender, Age, Mother’s age and education, Family structure and income, Dental caries, TDI, Malocclusion	**Impact on Overall ECOHIS** Presence TDI—PR: 1.70 (1.27–2.27)
Gomes et al.[25] English	2014	Brazil	843; 3–5	Cross-sectional	TDI (Andreasen)	OHRQoL as a dichotomous variable (ECOHIS)	Gender, Child’s age, Type of preschool, Mother’s schooling, household income, Parent’s/ guardian’s age, Number of residents in home, Birth order, Perception of general health, perception of oral health, Dental caries, TDI, malocclusion	**Impact on CIS** Presence TDI—PR: 1.41(1.16–1.71); **Impact on FIS** Presence TDI—PR: 1.00 (0.78–1.27)
Guedes et al.[31]English	2014	Brazil	478; 1–5	Cross-sectional	TDI (O’Brien)	Scores of OHRQoL (ECOHIS)	Gender, Skin color, Household income, Have visited a neighbor, TDI, Anterior open bite, Dental caries, Cultural community centers, Workers association	**Impact on Overall ECOHIS** Presence TDI—RR: 1.49 (1.3–1,8)
Viegas et al.[26] English	2014	Brazil	1632; 5–6	Cross-sectional	TDI (Andreasen)	OHRQoL as a dichotomous variable (ECOHIS)	Gender, Number of residents in household, Household income, Social Vulnerability Index (residence), Parents’/caregivers’ schooling, Parent’s/caregiver’s assessment of child’s oral health, Parent’s/caregiver’s assessment of child’s general health, TDI, Type of TDI, Number of teeth affected by TDI, toothache, Visits to dentist, Dental caries experience	**Impact on CIS** Presence TDI—PR: 1.03 (0.91–1.17); **Impact on FIS** Presence TDI—PR: 0.97 (0.84–1.12)
Abanto et al.[4] English	2015	Brazil	1215; 1–4	Cross-sectional	TDI (Glendor)	Scores of OHRQoL (ECOHIS)	Age, Gender, Mother’s age and education, Father’s age and education, Family structure, Number of children, Household income; Severity of TDI, Types of Malocclusion, Dental caries,	**Impact on Overall ECOHIS** Presence TDI—PR: 0.87 (0.64–1.18)
Firmino et al.[27] English	2015	Brazil	415; 3–5	Case-control	TDI (Andreasen)	OHRQoL as a dichotomous variable (ECOHIS)	Gender, Age, Household income, Type of preschool, Parent’s/caregiver’s age, Mother’s schooling, Perception of general health, Perception of oral health, Visits to dentist, Dental caries, Caries severity, TDI, Type of TDI	**Impact on CIS** Presence TDI—OR: 2.11 (1.23–3.62)
Vieira Andrade et al. [28] English	2015	Brazil	335; 3–5	Case-control	TDI (Andreasen)	OHRQoL as a dichotomous variable (ECOHIS)	Gender, Age, Household income, Type of preschool, Toothache, Dental visits, Parent’s/caregiver’s schooling, Number of children in family, Caregiver’s relationship to child, Dental caries, Malocclusion, Place of occurrence of TDI, Number of teeth affected by TDI, Type TDI	**Impact on CIS** Presence TDI—OR: 1.15 (0.66–2.03)

Based on the Newcastle-Ottawa methodological quality scale, the two case-control studies scored 7 out of a maximum of 9 points. Among the eight cross-sectional studies, seven achieved the maximum score of 4 points and one received a score of 3 points (Table 2). The two independent reviewers were in agreement with regard to all items on the scale.

**Table 2:** Quality assessment criteria for cross-sectional and case-control studies using Newcastle-Ottawa Scale for observational studies.

**Case-Control**									
	**A**	**B**	**C**	**D**	**E**	**F**	**G**	**H**	**Total**
Firmino et al. 2015 [27]	1	1	1	1	1	1	1	0	7/9
Vieira-Andrade et al. 2015 [28]	1	1	1	1	1	1	1	0	7/9
**Cross-Sectional**									
	**I**	**J**	**K**	**L**	**M**	**N**	**O**	**P**	**Total**
Aldrigui et al., 2011 [3]	0	1	1	1	0	0	0	0	3/4
Viegas et al., 2012 [23]	1	1	1	1	0	0	0	0	4/4
Siqueira et al., 2013 [24]	1	1	1	1	0	0	0	0	4/4
Kramer et al., 2013 [9]	1	1	1	1	0	0	0	0	4/4
Gomes et al., 2014 [25]	1	1	1	1	0	0	0	0	4/4
Guedes et al., 2014 [31]	1	1	1	1	0	0	0	0	4/4
Abanto et al., 2015 [4]	1	1	1	1	0	0	0	0	4/4
Viegas et al., 2014 [26]	1	1	1	1	0	0	0	0	4/4

A) Case definition; B) Representativeness of cases; C) Selection of Controls; D) Definition of controls; E) Comparability of cases and controls; F) Ascertainment of exposure; G) Same method of ascertainment for cases and controls; H) Non-response rate; I) Representativeness of exposed cohort; J) Selection of non-exposed cohort; K) Ascertainment of exposure; L) Demonstration that outcome of interest was not present at start of study; M) Comparability of cohorts on basis of design or analysis; N) Assessment of outcome; O) Follow-up long enough for outcome to occur; P) Adequacy of follow up of cohorts

**Fig 2:**
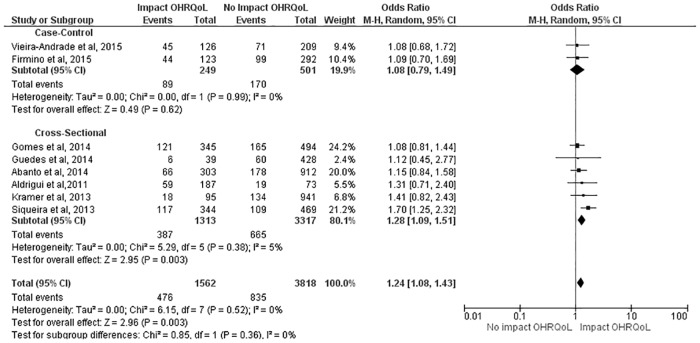
Subgroup analysis regarding effect of TDI on overall ECOHIS score.

The subgroup analysis allowed the description of an effect measure for each design as well as a combined measure. With regard to the overall score, no impact from TDI on OHRQoL was found in the case-control studies (OR: 1.08; 95% CI: 0.79 to 1.49), whereas a significant impact of TDI was found on the outcome in the cross-sectional studies (OR: 1.28; 95% CI: 1.09 to 1.51). In the combined measure of both designs, preschool children with TDI had a 24% greater chance of experiencing a negative impact on OHRQoL (OR: 1.24; 95% CI: 1.08 to 1.43) (Fig 2).

**Fig 3:**
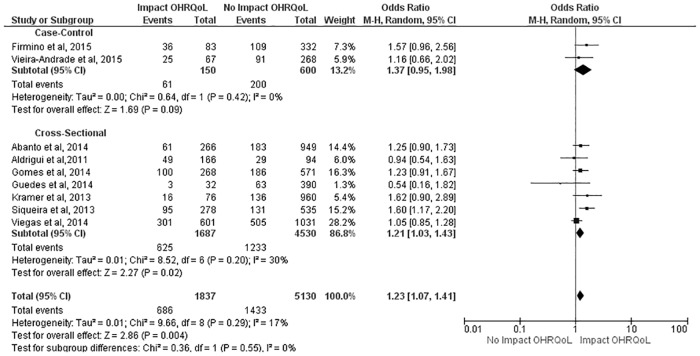
Subgroup analysis regarding effect of TDI on CIS scores.

**Fig 4:**
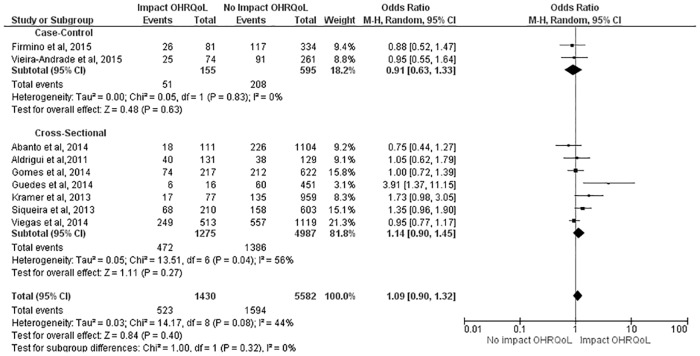
Subgroup analysis regarding effect of TDI on FIS scores.

Similar results were found with regard to the CIS, as no impact was detected in the case-control studies (OR: 1.37; 95% CI: 0.95 to 1.98), but preschool children in the cross-sectional studies had a 21% greater chance of experiencing a negative impact from TDI on OHRQoL (OR: 1.21; 95% CI: 1.03 to 1.43). The combined measure demonstrated that preschool children with TDI had a 23% greater chance of experiencing a negative impact on OHRQoL (OR: 1.23; 95% CI: 1.07 to 1.41) (Fig 3). With regard to the FIS, TDI had no negative impact on OHRQoL in either study design or in the combined measure (OR: 1.09; 95% CI: 0.90 to 1.32) (Fig 4).

## Discussion

The main finding of the present systematic review and meta-analysis is the negative impact of TDI on the OHRQoL of preschool children. To the best of our knowledge, this is the first systematic review to estimate such an effect. The evaluation of the methodological quality of the articles analyzed was extensive and allowed an accurate judgment for the summary and extrapolation of the findings.

All studies analyzed employed the ECOHIS to evaluate OHRQoL. This scale was designed and validated by Pahel et al. (2007) [19] and has been cross-culturally adapted to the Portuguese language [32]. The ECOHIS was developed based on the 36 items that compose the Child Oral Health Quality of Life Questionnaires [33]. The articles included in the present systematic review employed different cutoff points to classify impact on OHRQoL using the ECOHIS. Gomes et al. (2014) [25], Viegas et al. (2014) [26], Firmino et al. (2015) [27] and Vieira-Andrade et al. (2015) [28] considered responses of “never” or “hardly ever” as the absence of impact and at least one response of “occasionally”, “often” or “very often” as the presence of impact. However, Kramer et al. (2013) [9] and Siqueira et al. (2013) [24] considered “never” to be indicative of the absence of impact and included “rarely” among the other responses as indicative of the presence of impact. Aldrigui et al. (2011) [3], Viegas et al. (2012) [23], Abanto et al. (2015)[4] and Guedes et al. (2014) [31] did not define a cutoff point and therefore did not dichotomize the scores, but rather based their results on the quantitative variable.

The use of binary outcomes results in loss of statistical power and deciding on a cut-point may be arbitrary [34]. However, it determines results probably easier to understand and more meaningful for clinicians [34,35]. As mean differences on unfamiliar scales are of little use to most people [35], it is likely easier for clinicians and even patients to understand to what proportion the occurrence of an event increases the probability of impacting OHRQoL than to understand the difference in mean quality of life scores. Moreover, parents answer the ECOHIS questionnaire in a qualitative manner for each item (never, hardly ever, occasionally, often or very often) rather than quantitative. Thus, by describing proportion differences between groups, the investigation is directly reflecting the responses of the participants. As there is no universally accepted reference cutoff point for the ECOHIS, quality of life has been dichotomized in the present systematic review based on the original proposal by Pahel et al. (2007)[19].

The studies included in this review used different methods to diagnosis and classify TDI. Most employed the classification proposed by Andreasen and Andreasen et al. (2007) [22] and classified the preschool children as either “with TDI” or “without TDI”. A systematic review on the classificatory diagnostic method for TDI analyzed 164 articles published between 1936 and 2003, identifying 54 different classification systems [36]. Different classification criteria and different cutoff points can exert an influence on the results. Therefore, exposure was standardized in a dichotomous manner in the present investigation. A recent study described different magnitudes of impact of TDI on OHRQoL depending on whether children with only enamel fractures were or were not included in the exposed group [5]. In practice, it is less plausible for impact to be detected when children with the lowest exposure level are included. It is possible that the majority of parents/caregivers do not distinguish enamel fractures in young children, which could explain the lack of an impact from TDI [37]. Moreover, it is possible that the use of a cutoff point of a higher severity would detect an even greater impact on OHRQoL.

The main findings of the present study demonstrate that TDI exerts a negative impact on the OHRQoL of preschoolers based on the overall EOCHIS score as well as the score on the Child Impact Section. The literature describes that the domains most affected by TDI among preschool children are pain symptoms, function and self-image/social interaction [3,4,9]. Previous studies stress that a complicated TDI is expected to affect OHRQoL by producing discomfort, considering pulp involvement and/or tooth dislocation as well as the possibility of losing the affected tooth [3,4]. However, the results of the present meta-analysis, which summarized studies with similar methods and good methodological quality, leads to the conclusion that TDI exerts an impact on the quality of life of preschool children (overall ECOHIS and CIS scores) independently of the severity.

The present findings underscore the importance of the early diagnosis of TDI and the need for preventive measures to avoid tooth injuries from causing important adverse conditions in children and adolescents. Moreover, public health policies directed at reducing harm are needed, especially in regions for which human and/or material resources are scarce.

In contrast, TDI did not exert an impact on the Family Impact Section. Among the possible explanations for this finding is the high prevalence of enamel fractures in the surveys, for which fleeting discomfort on the part of the child may go undetected by parents/caregivers [5,23]. Moreover, TDI is a cumulative condition and it is possible that the adaptation of children to this condition leads to the attenuation of the impact of TDI on OHRQoL over time, thereby diminishing or even impeding the possibility of tooth injuries being detected by parents/caregivers [3].

Some methodological aspects of the present systematic review should be addressed. Different scales are used to determine the quality of studies included in systematic reviews. The Newcastle-Ottawa Scale for case-control studies and the same scale modified for cross-sectional studies were used in the present systematic review. This scale was developed to evaluate the quality of non-randomized studies (including case-control and cohort studies) with its design, content and ease of use directed toward the task of incorporating the evaluations of quality in the interpretation of the results of a meta-analysis [18]. Moreover, this scale is the most often employed in systematic reviews of observational studies. The modified Newcastle-Ottawa Scale was used for the cross-sectional studies, since there are no standardized, universally accepted scales for this type of design [38,39].

It should be stressed that, although the case-control design is hierarchically superior to the cross-sectional design, its main strengths—efficiency for rare outcomes and the retrospective approach [40]–do not apply to the case-control studies included in the present investigation, as both were nested in cross-sectional studies. Moreover, these studies exhibit the main bias found in the case-control design: the groups to be compared are constructed by the researcher and are not constituted naturally [40,41]. An additional problem and one that may at least partially explain the lack of an association between exposure and outcome is overmatching, which is a common bias in case case-control studies that biases the odds ratio toward 1 and diminishes the ability of a study to detect a significantly increased odds ratio [41]. However, the exclusion of the case-control studies would not have altered the main findings of the present systematic review.

The scores demonstrate the excellent methodological quality of the studies included. Both case-control studies received no score regarding the response rate, as neither article addressed this issue. Among the cross-sectional studies, only one received no score on the selection of the individuals, as the sample was a specific group treated at a university clinic and was not selected randomly from the community. The maximum score attributed to a cross-sectional study is 4 points, since the independent, blinded evaluation, comparability of cohorts, sufficient follow up of cohorts and adequacy of the follow-up period are specific to cohort studies.

In the present investigation, the probability of selection bias is small, as the search was performed in general databanks and allowed the detection of unpublished studies and grey literature, such as doctoral theses as well as abstracts presented at conferences and published exclusively on *Google Scholar*. Moreover, MESH terms and key words commonly used in articles published in the field were employed and manual searches of all references in the selected articles were performed for possible articles that were not detected in the electronic searches. All this care was taken to minimize the possibility of overlooking potentially eligible studies.

This systematic review and meta-analysis provides the first quantitative evidence regarding the significant impact of TDI on the OHRQoL of preschool children. The findings suggest that programs designed to reduce the occurrence of TDI in early childhood have the potential to contribute to OHRQoL. This includes educational actions and the mapping of situations of risk in the environments where children spend their time, such as the home, preschools and daycare centers [5]. The results of the present study do not indicate that all traumatic dental injuries in preschool children require treatment, as the impact on quality of life varies in accordance with the severity of the trauma [5]. As patient-related outcomes are poorly represented in dental traumatology [10], clinical trials should assess whether and how the treatment of traumatic dental injuries contributes to the oral health-related quality of life of preschool children and their families.

## Conclusion

There is a moderate quality of evidence suggesting a significant impact of TDI in the primary dentition on the OHRQoL of preschool children. The present findings indicate the need for TDI prevention and treatment programs in early childhood, including combating risk factors, the establishments of safe environments and prompt care.

## Supporting information

S1 Appendix Preferred Reporting Items for Systematic Reviews and Meta-Analyses (PRISMA). (PDF)

S2 Appendix PROSPERO registry. (PDF)
