# Supplementary material for: Impact of traumatic dental injuries on oral health-related quality of life of preschool children: A systematic review and meta-analysis
Source: PLoS One. 2017 Feb 28;12(2):e0172235. doi: 10.1371/journal.pone.0172235 (PMC5330474; doi:10.1371/journal.pone.0172235)
Supplement: S2 Appendix — (PDF) [file pone.0172235.s002.pdf]

PROSPERO International prospective register of systematic reviews

Review title and timescale

1 Review title
Give the working title of the review. This must be in English. Ideally it should state succinctly the interventions or exposures being reviewed and the associated health or social problem being addressed in the review.
Impact of Traumatic Dental Injuries on oral health-related quality of life of preschool children: systematic review and meta-analysis.

2 Original language title
For reviews in languages other than English, this field should be used to enter the title in the language of the review. This will be displayed together with the English language title.
English

3 Anticipated or actual start date
Give the date when the systematic review commenced, or is expected to commence.
12/05/2015

4 Anticipated completion date
Give the date by which the review is expected to be completed.
21/07/2016

5 Stage of review at time of this submission
Indicate the stage of progress of the review by ticking the relevant boxes. Reviews that have progressed beyond the point of completing data extraction at the time of initial registration are not eligible for inclusion in PROSPERO. This field should be updated when any amendments are made to a published record.

The review has not yet started ×

Review stage	Started	Completed
Preliminary searches	No	Yes
Piloting of the study selection process	No	Yes
Formal screening of search results against eligibility criteria	No	Yes
Data extraction	Yes	No
Risk of bias (quality) assessment	No	No
Data analysis	No	No

Provide any other relevant information about the stage of the review here.

Review team details

6 Named contact
The named contact acts as the guarantor for the accuracy of the information presented in the register record.
Tássia Borges

7 Named contact email
Enter the electronic mail address of the named contact.
tassia.s.borges@hotmail.com

8 Named contact address
Enter the full postal address for the named contact.
103 Joana Machado Street, Apt.302. Santa Maria, Rio Grande Do Sul.

9 Named contact phone number
Enter the telephone number for the named contact, including international dialing code.
(+55 55) 9914 7698.

10 Organisational affiliation of the review
Full title of the organisational affiliations for this review, and website address if available. This field may be completed

as 'None' if the review is not affiliated to any organisation.
Department of Pediatric Dentistry, Universidade Luterana do Brasil (ULBRA), Brazil

Website address:
http://www.ulbra.br/canoas

11 Review team members and their organisational affiliations
Give the title, first name and last name of all members of the team working directly on the review. Give the organisational affiliations of each member of the review team.

Title	First name	Last name	Affiliation
Dr	Tássia	Borges	Department of Pediatric Dentistry, Universidade Luterana do Brasil (ULBRA), Brazil
Dr	Fabiana	Vargas-Ferreira	Department of Pediatric Dentistry, Universidade Luterana do Brasil (ULBRA), Brazil
Professor	Paulo	Kramer	Department of Pediatric Dentistry, Universidade Luterana do Brasil (ULBRA), Brazil
Professor	Carlos	Feldens	Department of Pediatric Dentistry, Universidade Luterana do Brasil (ULBRA), Brazil

12 Funding sources/sponsors
Give details of the individuals, organizations, groups or other legal entities who take responsibility for initiating, managing, sponsoring and/or financing the review. Any unique identification numbers assigned to the review by the individuals or bodies listed should be included.
Study supported by the Brazilian Coordination of Higher Education, Ministry of Education (CAPES).

13 Conflicts of interest
List any conditions that could lead to actual or perceived undue influence on judgements concerning the main topic investigated in the review.
Are there any actual or potential conflicts of interest?
None known

14 Collaborators
Give the name, affiliation and role of any individuals or organisations who are working on the review but who are not listed as review team members.

Title	First name	Last name	Organisation details

Review methods

15 Review question(s)
State the question(s) to be addressed / review objectives. Please complete a separate box for each question.
Do traumatic dental injuries impact on oral health-related quality of life of preschool children?

16 Searches
Give details of the sources to be searched, and any restrictions (e.g. language or publication period). The full search strategy is not required, but may be supplied as a link or attachment.
There were no language or date of publishing restrictions. We searched the following electronic databases: A) PUBMED B) SCIENCE DIRECT C) WEB OF SCIENCE D) EMBASE E) SCOPUS F) GOOGLE SCHOLAR

17 URL to search strategy
If you have one, give the link to your search strategy here. Alternatively you can e-mail this to PROSPERO and we will store and link to it.

I give permission for this file to be made publicly available

Yes

18 Condition or domain being studied

Give a short description of the disease, condition or healthcare domain being studied. This could include health and wellbeing outcomes.

Oral diseases and disorders during childhood may have a negative impact in the quality of life of preschool children and their families. Traumatic dental injuries (TDI) are a common oral disorder in children, which is distressing experience on a physical level, but may also have an effect on their emotional and psychological levels, presenting a likely negative impact on their quality of life. However, studies about TDI and quality of life in preschool children are scarce and present controversial results. To the best of our knowledge, there is no systematic review or meta-analysis on Traumatic Dental Injuries and their impact on quality of life of preschool children. This systematic review and meta-analysis aims to assess the impact of traumatic dental injuries on oral health-related quality of life of preschool children.

19 Participants/population

Give summary criteria for the participants or populations being studied by the review. The preferred format includes details of both inclusion and exclusion criteria.

Inclusion: children aged zero to six years. Exclusion: individuals (> 6 years-old).

20 Intervention(s), exposure(s)

Give full and clear descriptions of the nature of the interventions or the exposures to be reviewed

This is a systematic review of observational studies and TDI is the main exposure of the study.

21 Comparator(s)/control

Where relevant, give details of the alternatives against which the main subject/topic of the review will be compared (e.g. another intervention or a non-exposed control group).

Not applicable

22 Types of study to be included initially

Give details of the study designs to be included in the review. If there are no restrictions on the types of study design eligible for inclusion, this should be stated.

Inclusion Criteria To be included in the review, a paper should have met the following criteria: (a) epidemiologic studies, without any restriction of design (such as cross-sectional, case-control, cohort studies and randomized clinical trials) that investigated the impact of TDI on the outcome in children aged zero to six years. Exclusion Criteria Review articles, case reports, expert opinions, studies involving the permanent dentition and guidelines and papers not using a validated instrument to assess the outcome were excluded from the review.

23 Context

Give summary details of the setting and other relevant characteristics which help define the inclusion or exclusion criteria.

24 Primary outcome(s)

Give the most important outcomes.

Ocurrence of impact on Oral Health-Related Quality of Life (OHRQoL) (yes/no) of preschool children.

Give information on timing and effect measures, as appropriate.

Prevalence Ratio (PR); Odds Ratio (OR)

25 Secondary outcomes

List any additional outcomes that will be addressed. If there are no secondary outcomes enter None.

occurrence of impact on Quality of life of the child (CIS) and on impact on Quality of life of the family (FIS).

Give information on timing and effect measures, as appropriate.

Prevalence Ratio (PR); Odds Ratio (OR)

26 Data extraction, (selection and coding)

Give the procedure for selecting studies for the review and extracting data, including the number of researchers involved and how discrepancies will be resolved. List the data to be extracted.

A data extraction spreadsheet was developed and two researchers collected the information independently. When disagreements occurred, discussion was made to obtain consensus, without the need for a third evaluator.

27 Risk of bias (quality) assessment

State whether and how risk of bias will be assessed, how the quality of individual studies will be assessed, and whether and how this will influence the planned synthesis.
The quality of the studies will be verified by two researchers (TSB and FVF), using the Newcastle-Ottawa Scale (NOS).

28 Strategy for data synthesis
Give the planned general approach to be used, for example whether the data to be used will be aggregate or at the level of individual participants, and whether a quantitative or narrative (descriptive) synthesis is planned. Where appropriate a brief outline of analytic approach should be given.
We will provide a narrative synthesis of the findings from the included studies, structured around, target population characteristic and type of outcome. The Comprehensive Meta-Analysis software program (Revman), will be used for the meta-analysis. Heterogeneity among the studies will be evaluated using I2 statistics. Meta-analysis will be conducted when I2 is below 50%, as values above 50% would indicate moderate to high heterogeneity, which may preclude meta-analysis.

29 Analysis of subgroups or subsets
Give any planned exploration of subgroups or subsets within the review. ‘None planned’ is a valid response if no subgroup analyses are planned.
If the data are available, subgroup analyses will be carried out to compare the influence of different cut-off point involving the outcome.

Review general information

30 Type of review
Select the type of review from the drop down list.
Epidemiologic

31 Language
Select the language(s) in which the review is being written and will be made available, from the drop down list. Use the control key to select more than one language.
English

Will a summary/abstract be made available in English?
No

32 Country
Select the country in which the review is being carried out from the drop down list. For multi-national collaborations select all the countries involved. Use the control key to select more than one country.
Brazil

33 Other registration details
Give the name of any organisation where the systematic review title or protocol is registered together with any unique identification number assigned. If extracted data will be stored and made available through a repository such as the Systematic Review Data Repository (SRDR), details and a link should be included here.

34 Reference and/or URL for published protocol
Give the citation for the published protocol, if there is one.
Give the link to the published protocol, if there is one. This may be to an external site or to a protocol deposited with CRD in pdf format.

I give permission for this file to be made publicly available
Yes

35 Dissemination plans
Give brief details of plans for communicating essential messages from the review to the appropriate audiences.
Do you intend to publish the review on completion?

36 Keywords
Give words or phrases that best describe the review. (One word per box, create a new box for each term)

dental injuries

dental trauma

traumatic dental injury

traumatic dental

tooth injuries

tooth fractures

quality of life

preschool

child

children

37 Details of any existing review of the same topic by the same authors
Give details of earlier versions of the systematic review if an update of an existing review is being registered, including full bibliographic reference if possible.

38 Current review status
Review status should be updated when the review is completed and when it is published.
Ongoing

39 Any additional information
Provide any further information the review team consider relevant to the registration of the review.

40 Details of final report/publication(s)
This field should be left empty until details of the completed review are available.
Give the full citation for the final report or publication of the systematic review.
Give the URL where available.
